# A Systematic Review of Phase II/III Trials of Hypofractionated versus Conventionally Fractionated Radiation Therapy in Stage III Non-Small Cell Lung Cancer Patients

**DOI:** 10.3390/cancers16193384

**Published:** 2024-10-03

**Authors:** May N. Tsao, Yee Ung, Patrick Cheung, Ian Poon, Alexander V. Louie

**Affiliations:** Odette Cancer Centre, Department of Radiation Oncology, University of Toronto, Toronto, ON M4N 3M5, Canada

**Keywords:** lung cancer, hypofractionated, radiation, systematic review

## Abstract

**Simple Summary:**

This structured review evaluated prospective trials that included non-metastatic advanced stage (Stage III) non-small cell lung cancer patients to determine if survival was different for high radiation dose per day (>2 Gy per day), given over a shortened number of radiation treatments (less than 30), known as hypofractionation, as compared to the usual standard radiation regimen, known as conventionally fractionated radiation therapy (2 Gy radiation dose, given once daily on weekdays for 30 daily treatments). There was no evidence that hypofractionation improves survival as compared to conventionally fractionated radiation therapy. Toxicity varied among the studies. Larger trials are needed to assess whether hypofractionated radiation is equivalent to conventional radiation. It is unclear whether the use of systemic therapy will improve survival outcomes with hypofractionated radiation and how the use of systemic therapy may negatively affect radiation toxicity with hypofractionation.

**Abstract:**

Introduction: This systematic review evaluated whether curative intent hypofractionated radiation therapy improved survival (primary endpoint) as compared to standard conventionally fractionated radiation therapy for stage III non-small cell lung cancer (NSCLC) patients. Toxicity was also examined as a secondary endpoint. Methods: Electronic bibliographic databases were searched from 1 January 1990 to 31 March 2024. Phase II and phase III trials were included to assess survival (primary outcome) and toxicity (secondary outcome) for newly diagnosed stage III NSCLC patients. Results: Eight phase II trials (*n* = 349 participants), 3 randomized phase II trials (*n* = 382 participants), and 5 randomized phase III trials (*n* = 811 participants), for a total of 1542 participants, were identified. The published trials were heterogeneous, with a wide variety of dose prescriptions. A wide range of survivals (median survival 13.6 months–42.5 months) and toxicities such as grade 3 or higher esophagitis (0–42%) and grade 3 or higher pneumonitis (0–18%) were reported. Conclusions: There is no level 1 evidence to date that suggests that any hypofractionated regimen (dose escalated or not) improves survival as compared to conventionally fractionated radiation. The published phase III trials have been powered for superiority (not equivalence) for the hypofractionated arm. Toxicity with hypofractionated regimens may be similar to conventionally fractionated regimens when normal tissue radiotherapy constraints are kept within tolerance limits. It is unclear how the use of systemic therapy may negatively affect radiation toxicity with hypofractionated radiation therapy.

## 1. Introduction

The standard radiation management for stage III non-small cell lung cancer (NSCLC) is conventionally fractionated radiation therapy [1,2]. Numerous trials have demonstrated that the standard dose fractionation of radiation for stage III NSCLC is 60 Gy in 2 Gy daily fractions given over 6 weeks. Radiobiological doses below 60 Gy were associated with more infield recurrences [3,4]. Dose escalation above 60 Gy with conventional fractionation has not demonstrated a survival benefit [5,6,7,8,9]. The current standard of care for eligible unresectable stage III NSCLC patients is concurrent platinum-based chemotherapy with conventionally fractionated radiation therapy (60 Gy) followed by durvalumab [10].

Whereas conventionally fractionated radiation therapy uses 1.8–2 Gy daily doses, hypofractionated radiotherapy uses a higher once daily dose (>2 Gy per day), reducing the overall treatment time and overall number of radiation fractions. Hypofractionation allows for reduced number of radiation treatment visits. Biological effective doses (BEDs) to the tumor target with hypofractionation may be similar to 60–66 Gy in 30-33 daily fractions or may be dose escalated. Guidelines exist for normal tissue constraints in the setting of conventionally fractionated chest radiation and for lung stereotactic body radiation therapy (SBRT). However, organ at-risk constraints for hypofractionated chest radiation have largely been extrapolated from BED calculations used in conventionally fractionated radiation therapy rather than clinical data from hypofractionated radiation therapy experience [11].

This systematic review evaluated whether curative intent hypofractionated radiation therapy (given with or without systemic therapy) improves survival (primary endpoint) as compared to standard conventionally fractionated radiation therapy (60 Gy in 2 Gy daily fractions given over 6 weeks) given with or without systemic therapy for stage III NSCLC patients. Toxicity was also examined as a secondary endpoint.

## 2. Methods

The protocol for this systematic review was prospectively registered on PROSPERO (PROSPERO 2022 CRD42022341893) and is available in full on the National Institute for Health and Care Research (NIHR) website: (https://www.crd.york.ac.uk/prospero/display_record.php?ID=CRD42022341893, date of registration 4 July 2022.

Phase II and phase III trials were included to assess survival (primary outcome) and toxicity (secondary outcome). For relevant trials, survival and toxicity outcomes between curative intent hypofractionated chest radiotherapy as compared to conventionally fractionated radiation therapy (with or without chemotherapy) were made. Published abstracts were excluded. Other altered fractionation schemes, such as hyperfractionated/accelerated radiation regimens, were excluded.

Curative intent chest radiation was defined as a radiotherapy regimen with a BED using α/β = 10 for tumor (BED10) of at least 70. This is the BED10 for 55 Gy in 20 fractions used commonly as a radical chest radiation regimen for NSCLC in the United Kingdom [12,13]. In comparison, BED10 for 60 Gy in 30 daily fractions is 72.

Published abstracts without full publication and publications in languages other than English were excluded.

The following electronic bibliographic databases were searched from 1 January 1990 to 31 March 2024: MEDLINE, EMBASE, The Cochrane Library (Cochrane Database of Systematic Reviews, Cochrane Central Register of Controlled Trials (CENTRAL), Health Technology Assessment Database, and the Web of Science (science and social science citation index). We restricted the included trials to full publications reported in English. Search results were assessed by 2 reviewers. Any disagreements were resolved through mutual discussion or through a third reviewer.

Appendix A lists the search strategy.

ClinicalTrials.gov was also searched for ongoing phase II or III hypofractionated trials in the management of newly diagnosed stage III non-small cell lung cancer participants.

The following data were extracted:

Study setting (single center, multi-center).

Participant demographics (stage group).

Intervention (radiation regimen).

BED with α/β ratio = 10 (BED10) was used to calculate tumor BED. Additionally, tBED10, which accounts for overall treatment time, was also calculated (15).

Systemic therapy (neoadjuvant, concurrent, adjuvant, and type).

Outcomes (survival, toxicity with a focus on pulmonary and esophageal toxicity, quality of life).

Quantitative synthesis for the primary outcome of survival using aggregate data were used if the included studies were sufficiently homogenous. For homogenous studies, overall survival was pooled using random-effects meta-analysis with risk ratios, 95% confidence intervals, and two-sided *p* values.

Toxicity was described using narrative synthesis.

Methodological quality (risk of bias) was assessed using ROBINS-1 (risk of bias in non-randomized studies of intervention) [14] and RoB 2 (revised tool for risk of bias in randomized trials) [15].

GRADE (Grading of Recommendations Assessment, Development, and Evaluation) was used for assessing the certainty of the evidence for the pooled phase III randomized controlled trials [16].

This report complies with the PRISMA (Preferred Reporting Items for Systematic Reviews and Meta-Analyses) 2020 checklist [17].

## 3. Results

**Figure 1:**
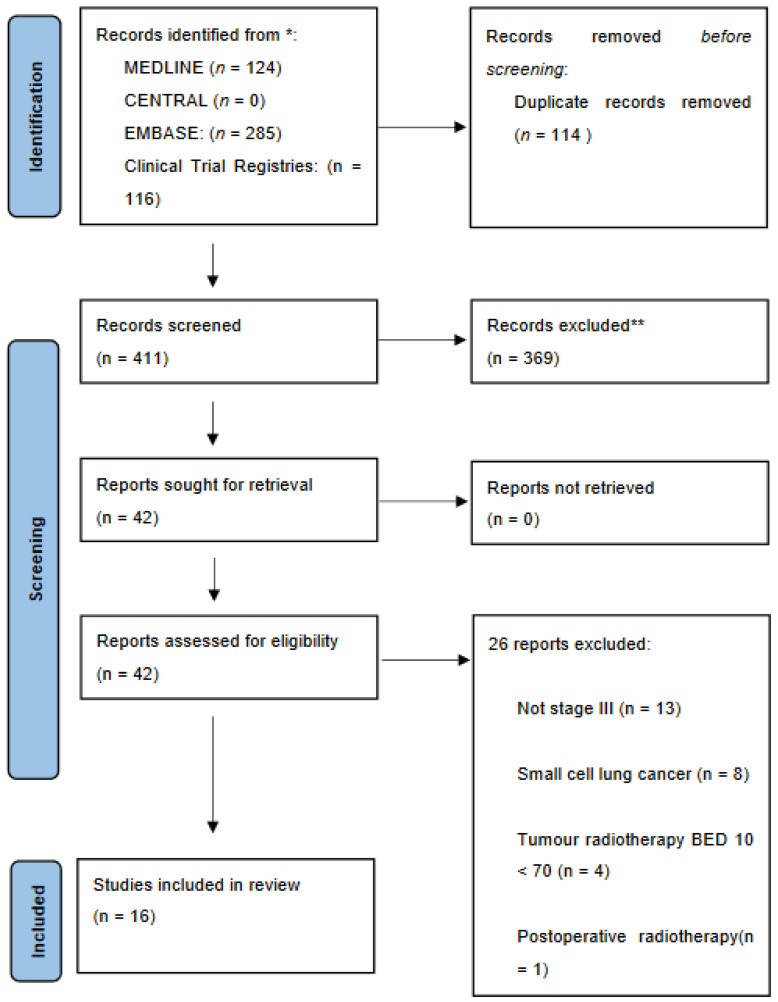
PRISMA 2020 Flow Diagram. BED10 = Biological Effective Dose α/β 10. Source: ref. [17].

There were 124, 0, and 285 publications identified through MEDLINE, CENTRAL (Cochrane reviews or Cochrane protocols), and EMBASE databases, respectively, during the period from 1 January 1990 to 31 March 2024. After duplicates and irrelevant studies were removed, there were a total of 42 full-text articles assessed for eligibility. Twenty-six records were excluded, which left 16 studies for qualitative and quantitative synthesis (Figure 1).

### 3.1. Phase II Trials

Eight phase II trials [18,19,20,21,22,23,24,25] were identified (Table 1). All were single-center studies except for two multicenter studies [20,25]. One of the multicentre studies [25] used proton radiation, whereas all other studies used photon radiation. Sample sizes ranged from 12 participants to 89 participants.

Using ROBINS-1 risk of bias assessment [14] for non-randomized studies, each of the included phase II trials scored moderate risk of bias. The included phase II trials were sound for non-randomized studies but are not comparable to well-performed randomized trials. Potential biases included the small sample size of these trials, resulting in a moderate risk of selection bias. Missing data in some of these studies may have also influenced outcomes. Although there was low risk in the reporting of survival, there may have been moderate risk in the reporting of toxicity.

None of the phase II trials reported on quality of life outcomes.

Radiation dose fractionation and chemotherapy regimens were heterogenous, such that results were not pooled. The BED10 for the radiation regimens ranged from 75–93.6 and tBED10 ranged from 72.5–91.1.

In addition, there was stage heterogeneity among the trials. Two trials [23,25] included stage II patients (as well as stage III patients). One trial included stage I-III participants [20].

Median survival was reported in 4 phase II trials [18,20,22,23]. In one of these trials, median survival was not reached at the highest radiation dose level (only 9 patients), with a median follow-up of 24.2 months [23]. Otherwise, median survival ranged from 13.6 months to 27 months [18,20,22].

Four trials reported one-year overall survival [19,21,24,25]. For these four trials, one-year survival ranged from 78.6% to 94.7%.

Acute grade 3 esophagitis ranged from none reported to 42%. All these phase II trials reported no acute grade 4 esophagitis [18,19,20,21,22,23,24,25]. One single-center phase II trial [20] reported a 4% risk of grade 5 (fatal) esophageal toxicity.

Grade 3 pneumonitis ranged from none to 28.6%. Seven of the included phase II trials reported no acute grade 4 pneumonitis [18,19,20,21,22,23,24]. The remaining trial [25] reported 1 patient out of 28 patients treated with proton hypofractionation who developed grade 4 pneumonitis. One single-center phase II trial [23] reported 2 (out of 9 patients) who experienced grade 5 (fatal) lung toxicity in the high-dose escalated hypofractionated arm.

### 3.2. Randomized Phase II Trials

Three randomized phase II trials [26,27,28,29] were identified (Table 2). All three were multi-center trials. One trial was closed early due to poor accrual [26]. Sample sizes ranged from 102–150 participants. Two trials included stage II NSCLC participants [26,28,29], in addition to stage III patients.

Using RoB 2 [15], the revised Cochrane risk of bias tool for randomized trials, each of the randomized phase II trials scored some concerns. Each phase II trial was judged to raise some concerns in at least one domain but not to be at high risk of bias for any domain. Each of these trials had some concerns for the risk of bias due to deviations from the intended intervention. As well, there were some concerns for the risk of bias due to missing outcome data. Although the risk of bias was low for the outcome of survival, there were some concerns for the risk of bias in the outcome measurement for toxicity and, in one trial, quality of life [27].

Quality of life was reported in only one trial [27}. Using the European Organisation for Research and Treatment of Cancer Quality Of Life questionnaire-Core30 (EORTC QLQC-30) and the quality of life Lung Cancer module (LC-14), the authors reported that the mean quality of life did not differ between the concurrent chemotherapy and hypofractionated radiation arm as compared to the neoadjuvant chemotherapy followed by hypofractionated radiation arm at 6 and 12 months [27].

Hypofractionated radiation dose fractionation schedules were heterogeneous, as were the chemotherapy regimens used, such that the results were not pooled. BED10 for the radiation regimens studied ranged from 70.1–107.7 and tBED10 ranged from 68.5–105.2.

Two randomized phase II trials [27,28,29] examined the use of hypofractionated radiation, comparing 2 different systemic therapy regimens.

The largest randomized phase II trial [26] reported on 150 participants, but this study was closed early due to poor accrual (target sample size 164). This trial also included Stage II participants (12% of included patients), with 88% of the included patients having stage III NSCLC. Hypofractionated radiation was given to the whole primary tumor versus hypofractionated radiation to the whole primary tumor with dose escalation to the PET subvolume [26].

Radiation dose fractionation and chemotherapy regimens as well as included patients (some stage II, in addition to stage III) were too heterogenous such that the results were not pooled. Median overall survival ranged from 18 months to 33 months. Grades 3 or higher acute esophagitis ranged from 6–11%. Grades 3 or higher pneumonitis ranged from 0–5.2%.

### 3.3. Randomized Phase III Trials

There were five phase III randomized trials [30,31,32,33,34,35] identified (Table 3). All were multicentre trials except for two [33,34,35].

The radiation regimens associated with BED10 ranged from 72–90 and tBED10 ranged from 68.1–83.3. Three phase III trials [31,32,33,34] randomized stage III NSCLC patients to either hypofractionated radiation versus 60 Gy in 30 daily fractions of conventional radiation. In two trials [31,33,34], all patients were ineligible for concurrent chemotherapy. In the other trial [32], patients enrolled received concurrent weekly paclitaxel and cisplatin.

Using RoB 2 [15], the revised Cochrane risk of bias tool for randomized trials, each of the randomized phase III trials scored some concerns. Each phase III trial was judged to raise some concerns in at least one domain but not to be at high risk of bias for any domain. Each of these trials had some concerns for the risk of bias due to deviations from the intended intervention. As well, there were some concerns for the risk of bias due to missing outcome data. Although the risk of bias was low for the outcome of survival, there were some concerns for the risk of bias in the outcome measurement for toxicity.

None of the included phase III trials reported on quality of life outcomes.

The oldest randomized trial [33,34] was undertaken in the 1980’s at a single center. This trial included 150 participants (97% had stage III NSCLC and 3% had stage IV NSCLC). In that era, patients were not given concurrent chemotherapy. Radiation alone, given as 60 Gy in 12 weekly fractions, was compared to 60 Gy in 30 daily fractions. There was no significant difference in 2-year survival (23% versus 29%, respectively). However, 55% of participants experienced moderate to severe acute radiation esophagitis in the conventional (60 Gy in 30 daily fractions) radiation arm.

A small single-center trial [35] randomized 97 Stage II and III NCSLC patients to 65 Gy in 26 daily fractions (using a concomitant boost technique) versus 70.8 Gy in 38 daily fractions (one volume). Both arms did not have chemotherapy. These patients either refused chemotherapy or were deemed unsuitable for chemotherapy. Survival outcomes were not reported. No treatment-related mortality was found. No grade 3 lung or esophageal toxicity was found in the concomitant boost arm. Two patients treated to 70.8 Gy in 38 daily fractions developed acute grade 3 lung toxicity.

The largest randomized phase III trial examining hypofractionation [30] enrolled 158 NSCLC participants, with the majority of patients having stage III disease (93%) to either hypofractionated radiation (66 Gy in 24 daily fractions) with concurrent cisplatin versus neoadjuvant gemcitabine and cisplatin for 2 cycles followed by the same hypofractionated regimen. Due to slow accrual, this study closed early. There was no significant difference in overall survival between the two arms. Acute grades 3–4 esophagitis was 14% in the radiation with concurrent cisplatin arm and 5% in the neoadjuvant chemotherapy followed by radiation arm. Grades 3–4 pneumonitis was 18% versus 14%, respectively.

The second largest phase III trial [31] randomized 103 participants to 60 Gy in 15 daily fractions versus 60 Gy in 30 daily fractions. All patients were ineligible for concurrent chemotherapy. This study was closed early because the interim analysis showed futility in reaching the primary endpoint of improved overall survival with the hypofractionated arm.

**Figure 2:**
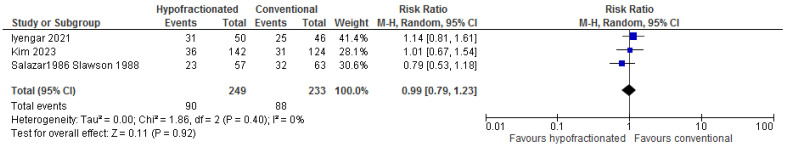
Forest plot (1 year overall survival for hypofractionated radiation versus conventional radiation) [31,32,33]. Review Manager 5.4.1 (RevMan 5.4.1) [Computer program]. Version 5.4.1. Copenhagen: The Cochrane Collaboration, 2020.

The 1 year overall survival results of the 3 phase III trials [31,32,33,34] comparing hypofractionated radiation therapy versus conventionally fractionated radiation therapy (60 Gy in 30 daily fractions) were pooled (Figure 2). Overall, there was no difference in overall survival with the use of hypofractionated radiation versus conventionally fractionated radiation (therapy RR 0.99 (95% confidence interval 0.79–1.23), *p* = 0.92). Using GRADE (16), the certainty of the evidence was moderate. It is noted, however, that the hypofractionated arms were different among the three trials: 60 Gy in 15 daily fractions [31], 60 Gy to GTV and 45 Gy to PTV in 25 daily fractions [32], and 60 Gy in 12 weekly fractions (5 Gy given once weekly [33,34]. In addition, 2 of the 3 trials did not allow concurrent chemotherapy [31,33,34]. The remaining trial [32] used concurrent weekly paclitaxel and cisplatin with the hypofractionated and conventional radiation arms.

Due to the lack of a conventional radiotherapy control arm (60 Gy in 30 daily fractions), the results of the remaining phase III trials [30, 35} were not pooled.

## 4. Discussion

The standard management for stage III NSCLC (in unresectable patients eligible for radical chest radiotherapy and systemic therapy) is concurrent platinum-based chemotherapy with conventionally fractionated radiation (60–66 Gy in 30–33 daily fractions) followed by adjuvant durvalumab [10].

Whereas conventionally fractionated radiation therapy uses 1.8–2 Gy daily doses, hypofractionated radiotherapy uses a higher once daily dose, which reduces the overall treatment time. Hypofractionation allows for reduced number of radiation treatment visits. Biologically effective doses to the tumor target with hypofractionation may be similar to 60–66 Gy in 30–33 daily fractions or may be dose escalated.

This systematic review identified 8 phase II trials (*n* = 349 participants), 3 randomized phase II trials (*n* = 382 participants), and 5 randomized phase III trials (*n* = 811 participants), for a total of 1542 participants treated with curative intent hypofractionated radiation therapy.

This report updates previous older systematic reviews (published in 2014 and 2018) on hypofractionated radiation therapy [36,37]. Unlike these other reviews, this updated report focuses on prospective phase II and III trials (excluding retrospective reports). In addition, only curative-intent hypofractionated regimens defined as a radiotherapy regimen with BED10 of at least 70 were included in this present report.

The published trials are heterogenous, with a wide variety of dose prescriptions, such as 55 Gy in 20 daily fractions to 80.6 Gy in 24 daily fractions. As well, some of the trials included stages I–IV NSCLC, in addition to Stage III patients. A wide range of survivals and toxicities were reported, which were not pooled due to the significant heterogeneity of the trials.

These trials also spanned a significant time from 1986–2024. During this time frame, optimal use of doublet platinum-based concurrent chemotherapy with conventionally fractionated radiation therapy was established. And more recently, adjuvant durvalumab has been shown to improve survival in eligible stage III NSCLC patients who are treated with concurrent chemoradiotherapy [10].

Furthermore, radiation techniques have evolved over this time period from 2D (dimensional) to 3D and now 4D planning. Radical radiation treatment delivery has changed from simple 2, 3, 4 field arrangements to 3D conformal radiation therapy to intensity modulated radiation therapy (IMRT)/volumetric modulated arc therapy (VMAT) to adaptive radiotherapy. The increased use of PET scans for staging and MRI brain imaging for staging may have resulted in stage migration for patients included in these trials. All these factors indicate that the outcomes for survival and toxicity from these trials should not be combined.

These trials do demonstrate that there is toxicity associated with hypofractionated radiation therapy with respect to grades 3 or higher radiation esophagitis and pneumonitis. However, the details of the dosimetry relating to these toxicity endpoints as well as the influence of systemic therapy on radiation-associated toxicity were not clearly elucidated. One phase III randomized trial reported that there were more patients with grade 3–4 acute esophagitis and pneumonitis with concurrent cisplatin and hypofractionated radiation (66 Gy in 24 daily fractions) as compared to sequential neoadjuvant gemcitabine and cisplatin followed by the same hypofractionated radiation [30].

The findings from the 5 published phase III randomized trials [30,31,32,33,34,35] indicate that any experimental curative intent hypofractionated radiation regimen (with or without systemic therapy) has not been compared to the current standard of concurrent chemoradiotherapy (60–66 Gy in 30–33 daily fractions) followed by adjuvant durvalumab.

All the 5 randomized phase III trials [30,31,32,33,34,35] do not demonstrate a survival benefit with the hypofractionated regimens studied with or without systemic therapy. For patients who are ineligible for concurrent systemic therapy, the use of 60 Gy in 15 daily fractions of radiation was not better for survival compared to 60 Gy in 30 daily fractions of radiation [31]. This trial was powered as a superiority trial (not equivalence). Grades 3–5 toxicity between the two arms in this trial were similar. Participants in this trial were planned using detailed target coverage requirements and normal tissue radiotherapy constraints based on this study protocol [31].

In addition, the multicentre Korean trial [32] failed to show superiority for the hypofractionated regimen of 60 Gy to the Gross Tumor Volume (GTV) with simultaneous 45 Gy to the Planning Target Volume (PTV) in 25 daily fractions as compared to 60 Gy in 30 daily fractions, with concurrent paclitaxel and cisplatin for both arms. Toxicity was similar between both arms.

In terms of normal tissue tolerance, a lung cancer consensus guideline [11] was developed for organs at risk constraints in the setting of hypofractionated chest radiation. If chemotherapy is not being delivered concurrently, the lung cancer consensus panel stated that 5940 to 7000/180 to 200 cGy conventional fractionation regimens or hypofractionated treatment (215–400 cGy/d) to a total dose of ≥5500 cGy to ≤6600 cGy may be appropriate. Detailed organs at risk constraints for various hypofractionated chest radiation regimens were provided in tabular form in the publication. These organs at risk constraint recommendations were largely based on panel consensus, extrapolation from BED calculations for organs at risk constraints in conventionally fractionated radiation therapy, and organs at risk constraints used in hypofractionated trial protocols.

Despite focusing this review on prospective phase II and III trials, there may have been some studies that were missed. The limitations of this review also include the wide range of time periods for which these trials accrued and followed patients. There may have been stage migration with the increasing use of PET scans for staging and brain MRIs over this timeframe. As well, systemic therapies associated with better survival outcomes and newer radiation technologies were developed. Further refinements with respect to radiation normal tissue tolerance based on dose volume characteristics have also evolved. However, there were insufficient details in the included trials regarding dose-volume characteristics related to toxicity and the impact of systemic therapy on toxicity. Except for 3 phase III trials [31,32,33,34], the other trials in this systematic review were too heterogeneous and were not pooled. Subgroup analyses were not conducted due to concerns regarding small sample sizes as well as variable treatments and patient characteristics in the included trials. Quality of life assessments were not reported except in one trial [27]. Individual patient data were also not acquired.

## 5. Future Directions

It remains to be seen whether hypofractionated approaches such as those listed in Table A1 and Table A2 will be successful in reaching target accrual. Further level 1 evidence is needed to assess the safety and efficacy of hypofractionated regimens given alone or with systemic therapy and immunotherapy as compared to conventionally fractionated radiation (with or without systemic therapy). In particular, a multi-center phase III trial (powered for non-inferior overall survival as the primary endpoint) is needed for stage III NSCLC patients comparing hypofractionated radiation therapy versus standard conventional radiation, with concurrent chemotherapy and adjuvant durvalumab given for both arms. For example, the ongoing NCT03331575 trial [38] is currently accruing unresectable stage III NSCLC patients to chemoradiation given as either hypofractionated radiation (60.5 Gy in 22 daily fractions) versus conventional radiation given as 60 Gy in 30 daily fractions. This trial is powered for the primary endpoint of overall survival with a sample size of 480 patients. In addition, prospective dose escalation studies using hypofractionation will allow for detailed evaluation of normal tissue dose volume characteristics (with and without systemic therapy) to help clarify radiation tolerance for the various hypofractionated radiation regimens. The evidence to date does not support survival benefits with the use of hypofractionated radiation therapy as compared to conventionally fractionated radiation therapy. Larger, high-quality trials (powered for equivalence) are needed to assess whether hypofractionated radiation is equivalent to conventional radiation. If future large phase III randomized trials show that hypofractionated radiation and conventionally fractionated radiation are equivalent for survival and similar in toxicity, hypofractionated radiation may be preferred for patient convenience and shortened overall treatment time. It is unclear whether the use of systemic therapy will improve survival outcomes with hypofractionated radiation and how the use of systemic therapy may negatively affect radiation toxicity with hypofractionation.

## 6. Conclusions

There is no compelling level 1 evidence to date that suggests that any hypofractionated regimen (dose escalated or not) improves survival as compared to conventionally fractionated radiation, with or without systemic therapy. There is insufficient evidence to indicate that hypofractionated radiation is equivalent for survival as compared to conventionally fractionated radiation, with or without systemic therapy. Toxicity with hypofractionated regimens may be similar to conventionally fractionated regimens when normal tissue radiotherapy constraints are kept within tolerance limits. It is unclear how the use of systemic therapy may negatively affect radiation toxicity.

## Figures and Tables

**Table 1:** Phase II trials.

Phase II Trials	Sample Size	Hypofractionated Radiation Therapy	Chemotherapy	Survival	Toxicity
Qiu 2021 [18] (single centre)	89 (all stage III)	51 Gy in 3 Gy daily fractions 3 week break 15–18 Gy in 3 Gy daily fractions boost BED10: 85.8–89.7 tBED10: 81.2–84.7	Weekly docetaxel and nedaplatin during radiation. Adjuvant systemic therapy not reported.	Median OS: 27 months	Esophagus: Grade 3 acute esophagitis: 16.9%. No grades 4–5 esophagitis. Lung: Grade 3 pneumonitis: 7.9%. No grades 4–5 pneumonitis.
Zhou 2023 [19] (single centre)	75 (all stage III)	40 Gy in 4 Gy daily fractions then 24–28 Gy in 4 Gy daily fractions boost BED10: 84.8–89.6 tBED10: 85.1–89.6	Weekly docetaxel and nedaplatin during radiation. Adjuvant systemic therapy not reported.	OS: 1 year 94.7%	Esophagus: Grade 2 acute radiation esophagitis: 26.7%. Grade 3 acute radiation esophagitis: 5.3%. No grades 4–5 esophagitis. Lung: Grade 2 acute pneumonitis: 17.3%. No grades 3–5 acute pneumonitis.
Cagney [20] 2018 (multicentre)	49 Stage I-II (35%) Stage III (65%)	60 Gy in 3 Gy daily fractions BED10: 78 tBED10: 76.3 or 66 Gy in 3 Gy daily fractions BED10: 85.8 tBED10: 83.6 or 72 Gy in 3 Gy daily fractions BED10: 93.6 tBED10: 91.1	Induction chemotherapy allowed, but no concurrent chemotherapy. Adjuvant systemic therapy not reported.	Median OS: 13.6 months	Esophagus: Grade 5 esophageal toxicity: 4%. Lung: Grades 2 or higher pneumonitis: 12%.
Katsuta [21] 2021 (single centre)	36 (all stage III); (Closed early due to slow accrual)	60–70 Gy in 2.5 daily fractions BED10: 75–87.5 tBED10: 72.5–84.7	Concurrent platinum-based doublet chemotherapy with radiation. No patient received adjuvant durvalumab.	1 year OS: 88.9%	Esophagus: Grade 3 esophagitis: 2.8%. No grades 4–5 esophagitis. Lung: Grade 3 pneumonitis: 8.3%. No grades 4–5 pneumonitis.
Casas [22] 2011 (single centre)	32 (all stage III)	61.64 Gy (1.8 Gy large fields and boost 0.88 Gy) in 23 daily fractions BED10: 78.2 tBED10: 75.7	Neoadjuvant paclitaxel and carboplatin with concurrent weekly paclitaxel and radiation. 2 cycles of adjuvant paclitaxel and carboplatin.	Median OS: 16.9 months	Esophagus: Grade 2 esophagitis: 28.1%. Grade 3 esophagitis: 6.2%. No grades 4–5 esophagitis. Lung: No grades 3–4 lung toxicity. Grade 5 lung toxicity: 3.1%.
Wu [23] 2024 (single centre)	28 Stage II (64%) Stage III (36%)	40 Gy in 4 Gy daily fractions then: Adaptive SBRT boost to residual PET avid disease: 25 Gy in 5 Gy daily fractions (low) BED10: 82.9 tBED10: 82.2 OR 30 Gy in 5 Gy daily fractions (intermediate) BED10: 84.2 tBED10: 83.1 OR 35 Gy in 5 Gy daily fractions (high) BED10: 90.4 tBED10: 89.2	Concurrent weekly carboplatin and paclitaxel. Adjuvant systemic therapy at the discretion of the treating physician.	Overall median: 25.9 months Low dose: 15.3 months Intermediate dose: 42.5 months High dose: Not reached	Esophagus: Grades 3 or higher esophageal toxicity: Low dose: 4% Intermediate dose: 0% High dose: 0% Lung: Grades 3 or higher lung toxicity: Low dose: 4% Intermediate dose: 0% High dose: 7% (2 patients grade 5)
Ren [24] 2016 (single centre)	12 (all stage III) Closed early due to slow accrual	69 Gy in 3 Gy daily fractions BED10: 89.7 tBED10: 87.2	Concurrent viorelbine and carboplatin OR Concurrent paclitaxel and cisplatin Adjuvant systemic therapy not reported	1 year OS: 78.6%	Esophagus: Grade 3 radiation esophagitis: 42%. No grades 4–5 esophagitis. Lung: Grade 3 radiation pneumonitis: 17%. No grades 4–5 pneumonitis.
Hoppe [25] 2022 (multicentre)	28 (Closed early due to slow accrual) Stage II (21%) Stage III (79%)	Protons: 60 Gy RBE at 2.5 Gy RBE daily fractions; 60 Gy RBE at 3 Gy RBE daily fractions; 60.01 Gy RBE at 3.53 Gy RBE daily fractions 60 Gy RBE at 4 Gy RBE daily fractions	Concurrent platinum-based doublet chemotherapy with radiation; Post-radiation chemotherapy or durvalumab optional.	1 year OS: 89%	Esophagus: No grade 3 or higher esophagitis. Lung: Grade 3 or higher lung toxicity: 14%.

BED10 = Biological Effective Dose alpha/beta 10 for tumor; Gy = Gray; NR = not reported; OS = overall survival; PET = positron emission tomography; PFS = progression-free survival; RBE = relative biological effectiveness; SBRT = stereotactic body radiation therapy; tBED10 = BED10 taking into account overall treatment time.

**Table 2:** Phase II randomized trials.

Randomized Phase II Trials	Sample Size	Arm 1	Arm 2	Survival	Toxicity
Cooke [26] 2023 (multicentre)	150 Stage II (12%) Stage III (88%) (Closed early due to slow accrual)	Dose escalation to whole primary tumor: Mean dose to PTV (IQR): 77 Gy (74.2–80.6 Gy) In 24 daily fractions; BED10: 97.5–107.7 tBED10: 95–105.2 Concurrent or sequential chemotherapy or radiation alone; Consolidation chemotherapy not allowed.	Dose escalation to PET subvolume Mean dose to PTV (IQR): 74.2 Gy (72.3–77.8 Gy) Mean dose to PET boost subvolume: 83.3 Gy (78–90.1 Gy) In 24 daily fractions; BED10: 103.3–123.8 tBED10: 100.9–121.3 Concurrent or sequential chemotherapy or radiation alone; Consolidation chemotherapy not allowed.	Median OS for both arms: 18 months	Overall acute grade 3 or higher for both arms: Dysphagia/ Esophagitis: 11% Dyspnea: 7% Radiation pneumonitis: 4%
Maguire [27] 2014 (multicentre)	130 All Stage III	55 Gy in 20 daily fractions with concurrent cisplatinum and vinorelbine BED10: 70.1 tBED10: 68.5	Neoadjuvant cisplatinum and vinorelbine; radiotherapy 55 Gy in 20 daily fractions to start 4 weeks after day 1 of the final cycle of chemotherapy BED10: 70.1 tBED10: 68.5	Median OS: Arm 1: 24.3 months Arm 2: 18.4 months (NS)	Grade 3 or higher esophagitis: Arm 1: 8.8% Arm 2: 8.5% (NS) Grade 3 or higher pneumonitis: Arm 1: 3.1% Arm 2: 5.2% (NS)
Walraven [28] 2016, van den Heuvel [29] 2014 (multicentre)	102 Stage II (8%) Stage III (92%)	66 Gy in 2.75 Gy daily fractions with concurrent cisplatin and no planned adjuvant systemic therapy BED10: 84.2 tBED10: 81.7	66 Gy in 2.75 Gy daily fractions with concurrent cisplatin and cetuximab and no planned adjuvant systemic therapy BED10: 84.2 tBED10: 81.7	Median OS: Arm 1: 33 months Arm 2: 30 months (NS)	Late grade 3 or worse lung toxicity: Arm 1: 0% Arm 2: 4% Late grade 3 or worse esophageal toxicity: Arm 1: 6% Arm 2: 8%

BED10 = Biological Effective Dose alpha/beta 10 for tumor; Gy = Gray; IQR = interquartile range; NR = not reported; NS = not statistically significant; OS = overall survival; PET = positron emission tomography; PTV = planning target volume; tBED10 = BED10 taking into account overall treatment time.

**Table 3:** Randomized phase 3 trials.

Randomized Phase III Trials	Sample Size	Arm 1	Arm 2	Survival	Toxicity
Belderbos [30] 2007 (multicentre)	158 Stage I (2%) Stage II (4.5%) Stage III (93%) Unknown (0.5%) (Closed early due to slow accrual)	66 Gy in 24 daily fractions (concurrent with cisplatin with no planned adjuvant systemic therapy) BED10: 84.2 tBED10: 81.7	2 cycles gemcitabine and cisplatin followed by 66 Gy in 24 daily fractions with no planned adjuvant systemic therapy BED10: 84.2 tBED10: 81.7	Median OS: Arm 1: 16.5 months Arm 2: 16.2 months (NS)	Acute esophagitis grades 3–4: Arm 1: 14% Arm 2: 5% Late grade 3 esophagitis: Arm 1: 4% Arm 2: 3% Pneumonitis grades 3–4: Arm 1: 18% Arm 2: 14%
Iyengar [31] 2021 (multicentre)	103 (after approximately half the target sample reached, planned interim analysis suggested futility in reaching primary endpoint, leading to study closure) Stage I (1%) Stage II (23%) Stage III (74%) Stage IV (2%)	60 Gy in 15 daily fractions (all patients ineligible for concurrent chemoradiation; 8% received systemic therapy before radiation and 26% received systemic therapy after radiation) BED10: 84 tBED10: 83.3	60 Gy in 30 daily fractions (all patients ineligible for concurrent chemoradiation; 6.5% received systemic therapy before radiation and 37% received systemic therapy after radiation) BED10: 72 tBED10: 68.1	1 year OS: Arm 1 37.7% Arm 2 44.6% *p* = 0.29	Grades 3–5 toxic effects attributable to radiation: Arm 1: 18 patients Arm 2: 19 patients (NS)
Kim [32] 2023 (multicentre)	303 (all stage III)	60 Gy to GTV and 45 Gy to PTV in 25 daily fractions (concurrent weekly paclitaxel and cisplatin with no planned adjuvant systemic therapy) BED10: 74.4 tBED10: 71.8	60 Gy in 30 daily fractions (concurrent weekly paclitaxel and cisplatin with no planned adjuvant systemic therapy) BED10: 72 tBED10: 68.1	Median OS: Arm 1: 27 months Arm 2: 26 months (NS)	No significant difference in grades 3 or higher radiation pneumonitis or radiation esophagitis; Cumulative 1 year grades 3 or higher esophagitis: Arm1: 2% Arm 2: 6% Cumulative 1 year grades 3 or higher pneumonitis: Arm 1: 3% Arm 2: 8%
Salazar [33] 1986/Slawson [34] 1988 (single center)	150 Stage III (97%); Stage IV (3%)	60 Gy in 12 weekly fractions (5 Gy given once weekly) BED10: 90 tBED10: 77.3 (with no concurrent chemotherapy and no planned adjuvant systemic therapy)	60 Gy in 30 daily fractions BED10: 72 tBED10: 68.1 (with no concurrent chemotherapy and no planned adjuvant systemic therapy)	2 year survival Arm 1: 29% Arm 2: 23% (NS)	Moderate to severe acute radiation esophagitis: Arm 1: 4% Arm 2: 55% Symptomatic pneumonitis: Arm 1: 1 patient Arm 2: none
Sun [35] 2000 (single center)	97 Stage I (5%); Stage II (21%); Stage III (74%)	65 Gy in 26 daily fractions (large field 1.8 Gy with concomitant boost 0.7 Gy) BED10: 81.3 tBED10: 78.6	70.8 Gy in 38 daily fractions BED10: 84 tBED10: 79.8	Not reported	Acute grade 3 lung toxicity: Arm1: none Arm 2: 2 patients Acute grade 3 esophagitis: Arm 1: none Arm 2: none No treatment-related mortality

BED10 = Biological Effective Dose alpha/beta 10 for tumor; Gy = Gray; NR = not reported; NS = not statistically significant; OS = overall survival; PET = positron emission tomography; PTV = planning target volume; tBED10 = BED10 taking into account overall treatment time.

## Appendix A

Search strategy:

1. Exp Lung Cancer/
2. Radiotherapy/
3. (radiotherapy or radiat *).mp
4. 2 or 3
5. 1 and 4
6. Hypofractionat *
7. 5 and 6
8. randomized controlled trial.pt.
9. controlled clinical trial.pt
10. randomized.ab.
11. randomly.ab
12. trial.ab.
13. phase 2.ab
14. phase II.ab
15. or/8–14
16. 7 and 15
17. Limit 18 to yr = “1990-”
18. key:
19. Exp = Explode
20. Mp = title, original title, abstract, subject heading, name of substance, registry word, unique identifier
21. Pt = publication type
22. ab = abstract

**Table A1:** Selected ongoing phase II trials of hypofractionated radiation therapy for stage III NSCLC.

Phase II Trials	Sample Size	Intervention	Primary Endpoint	Secondary Endpoint
Thoracic radiotherapy plus durvalumab in elderly and/or frail NSCLC stage III patients unfit for chemotherapy (TRADE-hypo). (NCT04351256) [39]	88	55 Gy/20 daily fractions/5 days a week	Grade 3 or higher pneumonitis Overall response rate at 12 weeks	Adverse events Overall survival Quality of life
Hypofractionated 3-dimensional radiation therapy in treating patients with newly diagnosed stage I, stage II, or stage III non-small cell lung cancer. ICORG 99–09. (NCT 00955175) [40]	60	Group 1: 72 Gy/24 daily fractions/5 days a week Group 2: 66 Gy/22 daily fractions/5 days a week Group 3: 60 Gy/20 daily fractions/5 days a week	Radiation toxicity	Radiological tumor response at 3 months Thoracic freedom from progression
Comparing hypo-fractionated intensity-modulated radiation therapy to standard-fractionated IMRT along with chemotherapy and immunotherapy for non-small cell lung cancer. (NCT04992780) [41]	50	Hypofractionation: 62.5 Gy in 25 daily fractions/5 days a week Standard fractionation: 60 Gy in 30 daily fractions/5 days a week	Locoregional control	Acute and late toxicity Progression-free survival Overall survival Quality of life
Personalized Accelerated ChEmoRadiation (PACER) for Lung Cancer: Protocol for a Bayesian Optimal Phase I/II Trial NCT06080061 [42]	45	60 to 66 Gy in 30, 25, or 20 fractions depending on the ability to meet constraints to key organs at ris, including the lungs, heart, and esophagus.	High-grade pulmonary, esophageal, and cardiac toxicity	Local control, progression-free survival, overall survival, feasibility of automated treatment planning workflow

Gy = Gray; ICORG = Irish Cooperative Oncology Research Group; IMRT = Intensity Modulated Radiation Therapy; NCT = National Clinical Trial; NSCLC = non-small cell lung cancer.

**Table A2:** Selected ongoing phase III trial of hypofractionated radiation therapy for stage III NSCLC.

Phase III Trials	Sample Size	Intervention	Primary Endpoint	Secondary Endpoint
Hypofractionated versus conventionally fractionated concurrent chemoradiation for unresectable stage III NSCLC. (NCT03331575) [38]	480	Hypofractionated radiotherapy: 60.5 Gy in 22 daily fractions/5 days a week with concurrent chemotherapy Conventional radiotherapy: 60 Gy in 30 daily fractions/5 days a week with concurrent chemotherapy	Overall survival	Disease-free survival Locoregional control Distant metastasis-free survival Adverse events

Gy = Gray; NCT = National Clinical Trial; NSCLC = non-small cell lung cancer.
